# Self-reported willingness to share political news articles in online surveys correlates with actual sharing on Twitter

**DOI:** 10.1371/journal.pone.0228882

**Published:** 2020-02-10

**Authors:** Mohsen Mosleh, Gordon Pennycook, David G. Rand

**Affiliations:** 1 Sloan School of Managment, Massachusetts Institute of Technology, Cambridge, Massachusetts, United States of America; 2 Hill/Levene Schools of Business, University of Regina, Regina, Saskatchewan, Canada; 3 Department of Brain and Cognitive Sciences, Massachusetts Institute of Technology, Cambridge, Massachusetts, United States of America; West Pomeranian University of Technology, POLAND

## Abstract

There is an increasing imperative for psychologists and other behavioral scientists to understand how people behave on social media. However, it is often very difficult to execute experimental research on actual social media platforms, or to link survey responses to online behavior in order to perform correlational analyses. Thus, there is a natural desire to use self-reported behavioral intentions in standard survey studies to gain insight into online behavior. But are such hypothetical responses hopelessly disconnected from actual sharing decisions? Or are online survey samples via sources such as Amazon Mechanical Turk (MTurk) so different from the average social media user that the survey responses of one group give little insight into the on-platform behavior of the other? Here we investigate these issues by examining 67 pieces of political news content. We evaluate whether there is a meaningful relationship between (i) the level of sharing (tweets and retweets) of a given piece of content on Twitter, and (ii) the extent to which individuals (total N = 993) in online surveys on MTurk reported being willing to share that same piece of content. We found that the same news headlines that were more likely to be hypothetically shared on MTurk were also shared more frequently by Twitter users, r = .44. For example, across the observed range of MTurk sharing fractions, a 20 percentage point increase in the fraction of MTurk participants who reported being willing to share a news headline on social media was associated with 10x as many actual shares on Twitter. We also found that the correlation between sharing and various features of the headline was similar using both MTurk and Twitter data. These findings suggest that self-reported sharing intentions collected in online surveys are likely to provide some meaningful insight into what content would actually be shared on social media.

## Introduction

Social media platforms such as Facebook and Twitter are now frequented by billions of people worldwide. It is no surprise, then, that there has been an increasing interest in investigating how people behave on social media, with many recent papers using sharing data from online platforms as real-world tests of a variety of important psychological theories [1–4]. There are at least two important ways that these sorts of studies different from more traditional psychological studies conducting in lab or using online surveys.

First, studies using social media sharing data are typically observational in nature. This makes it hard to draw clear causal inferences about the relationships observed. It also largely excludes an entire range of studies aimed at exploring the impact of experimentally manipulating various features of the content or environment–most notably, studies trying to develop interventions to address problems on social media such as misinformation and hate speech (Although it is possible to conduct field experiments on social media platforms [5–7] they are technically challenging and can raise ethical issues.).

Second, studies using social media data are often restricted in terms of what information is observable about subjects beyond their sharing behavior. This poses a major challenge for research that is focused on individual differences (and that therefore may not be concerned with the lack of randomization discussed above). Privacy concerns typically prevent social media platforms from making demographic or other individual difference data available to researchers. The same concerns also often interfere linking data from more traditional surveys with respondents’ social media accounts. Such linking is, for example, a violation of the terms of service on Amazon’s Mechanical Turk (MTurk)–a frequent source for participants in psychological research [8–11].

It would therefore be desirable to be able to investigate social media sharing in more traditional lab or online survey settings, rather than on-platform–and psychologists are increasingly engaging in such studies. For example, studies on fake news and misinformation have presented individuals with news headlines and asked online participants to indicate if they would be willing to share them on social media [12–16]. It is unclear, however, whether responses to such ‘hypothetical’ self-report social media sharing questions reliably tracks actual behavior online.

There are at least three reasons to expect that self-reported sharing intentions may not be a good proxy of sharing on-platform. First, individuals may lack insight into their own sharing behavior, such that their self-report of whether they would share content in the context of a survey might diverge from their actual decisions about what to share while on social media [17]. Second, the individuals who sign up for psychology (and otherwise) studies on platforms like MTurk may differ from individuals who frequent social media websites in meaningful ways, such that results observed on MTurk would not provide a useful guide to behavior on-platform. Third, sharing decisions on-platform may be influenced by who else in your network you have seen like, share, or comment on a headline–information which is not available on MTurk.

Empirical evidence evaluating these concerns is, to our knowledge, largely lacking. One somewhat-related piece of evidence comes from [18], who examined overall rates of sharing (rather than likelihood of sharing specific articles), and compared self-reports to actual behavior on Twitter and Facebook during the 2016 U.S. presidential election by matching survey data with social media profiles. They found that self-reports of tweet frequency showed a strong correlation with actual tweet frequency on Twitter (overall, *r* = .47; specifically political content, *r* = .45), as well as an actual quantitative agreement (e.g. people who said they tweet once a day actually tweeted on average 1.23 times per day); and self-reports of Facebook sharing frequency also correlated with actual sharing frequency (overall, *r* = .38; political, *r* = .32), although quantitatively people tended to substantially over-estimate their frequency of sharing. These data do not speak directly to our question of interest, however, as they examine retrospective description of aggregate behavior, whereas we are interested in participants’ forward-looking predictions about their future behavior.

In the current work, therefore, we investigate the extent to which social media sharing intentions elicited on MTurk provide insight into actual sharing on social media. In particular, we conduct our analysis at the headline level, and therefore avoid any privacy-related issues that may arise by conducting the analyses at the individual level. For a set of political news headlines, we compare the fraction of MTurk workers who say they would consider sharing the headline on social media with the number of times the headline was actually shared on Twitter (we focus on Twitter because of its open Application Programming Interface, which makes most sharing publicly observable). Our investigation is premised on the following claim: if self-report sharing intentions on MTurk provide useful signal about actual sharing behavior of the broader public, then the headlines that MTurkers say they would share more should actually receive much shares on Twitter. Here, we test this prediction.

## Materials and methods

### Sharing intentions on MTurk

To assess MTurk sharing intentions, we analyzed sharing data from the control conditions of the two previous experiments of ours in which participants indicated their willingness to share a series of actual news headlines from social media related to American politics [12, 13] (These are the only two such studies we have collected with clean sharing data. Our other studies that elicited sharing data, [15, 16], Study 1 of [12], or Study 3 of [13], are problematic as the sharing intention elicitation in these studies was confounded by asking people to rate the accuracy of each news headline immediately before indicating their willingness to share, therefore inducing a greater attention to accuracy than which is likely to be the case “in the wild”.). In each experiment, veracity was crossed with political leanings. Half of the headlines were false, based on fact-checking from Snopes.com, and half were true. Half of the headlines were Pro-Democratic and half were Pro-Republican, based on pre-testing. By crossing veracity with partisan leanings, these studies provide a fairly wide range of political news content that samples (albeit quite sparsely) from different areas of the space of potential headlines.

#### Participants

We had data for 505 participants (*M*_age_ = 36.6; 220 female, 272 male, 1 transgender female, 1 trans/non-binary, 3 preferred not to answer; 58.4% Democrats, 40% Republicans, 1.6% didn’t respond to our political ideology question; all of whom met the study’s eligibility requirements of having either a Twitter or a Facebook account and being willing to consider sharing political news on social media) from [12] and for 488 participants (*M*_age_ = 37; 249 female, 232 male, 7 did not answer the gender question; 65.6% preferred Hillary Clinton over Donald Trump in a forced choice) from [13]; all were United States residents recruited using MTurk.

#### Materials

As noted, participants were presented with a series of news headlines and asked whether they would consider sharing them on social media. The headlines were presented in ‘Facebook format’ (for example, “BREAKING NEWS: Hillary Clinton Filed For Divorce In New York Courts–The USA-NEWS, Bill Clinton just got served—by his own wife. At approximately 9:18 am on Thursday, attorneys for Hillary Rodham Clinton filed an Action For Divorce with the Supreme Court of …. THEUSA-NEWS.COM”; this is a false headline). In [12], participants were asked specifically about whether they would share the headlines on Facebook: “If you were to see the above article on Facebook, would you consider sharing it?” (response options: no, yes). In [13], participants were asked more generally about sharing the content online: “Would you consider sharing this story online (for example, through Facebook or Twitter)?” (response options: no, maybe, yes; scored as no = 0, maybe or yes = 1).

There were a total of 64 headlines that were presented to participants in [12], Study 2; however, each participant only made judgments for 32 headlines. Of the 64 headlines, 36 were false headlines (12 Pro-Democratic, 24 Pro-Republican) and 28 were true headlines (12 Pro-Democratic, 16 Pro-Republican). The headlines were selected from previous pretests because they were relatively ‘timeless’ in the sense that, although they were obtained between 2016 and 2018, they could have very well been from when the study was run (February 15^th^, 2019). The full set of headlines for [12] can be found on OSF: https://osf.io/b5m3n/.

[13], Experiment 2, used a somewhat overlapping set of 24 headlines (75 unique headlines across the two datasets), half of which were false (6 Pro-Democratic, 6 Pro-Republican) and half true (6 Pro-Democratic, 6 Pro-Republican). Participants only provided sharing intentions for half of the headlines. The headlines were selected in the same way as in [12], except they were not specifically intended to be “timeless”. The full set of headlines for [13] can be found on OSF: https://osf.io/txf46/.

#### Procedure

The instructions leading up to the sharing intentions task were somewhat different for the two experiments. In [12], participants were given the following instructions prior to completing the news sharing task: “You will be presented with a series of news headlines from 2016–2018 (32 in total). We are interested in whether you would be willing to share the story on social media (for example, on Facebook and/or Twitter—if the answer is ‘yes’ for one of the platforms but not the other, please respond with ‘yes’). Note: The images may take a moment to load. Also, the survey will auto-advance when you select an option for the news headlines.” This was followed by: “75% of the headlines in the study have been checked for accuracy using the professional fact-checking website Snopes.com. Snopes is the oldest and largest fact-checking site online, investigating the truth of urban legends, hoaxes, memes, and rumors on the internet for more than 25 years. Snopes has been independently verified by the International Fact-Checking Network (IFCN), which lists its core principles as: ‘non-partisanship and fairness, transparency of sources, transparency of funding and organization, transparency of methodology, and open and honest corrections policy.’”

In [13], participants were given only the following in terms of instructions prior to the task: “You will be presented with a series of actual news headlines from 2016 (12 in total). We are interested in whether you would be willing to share the story on social media (such as Facebook or Twitter). Please take your time and read the headlines. Note: The images may take a moment to load.”

To calculate our MTurk sharing variable, we calculated the fraction of participants saying they would share each headline in each of the two studies. For headlines that appeared in both studies, we then averaged across subjects in both studies.

### Actual sharing on Twitter

To determine the extent of actual sharing for each of the headlines, we used the Twitter search feature and searched for tweet IDs of all tweets between January 1, 2016 and March 5, 2019 that contained all of the words in a given headline. This time-period covers the first appearance of the earliest headlines in our data, and goes well beyond the first appearance of the most recent headline. Using this approach, we found at least one tweet for 68 of the 75 unique headlines across the two experiments. We then used the Twitter API to retrieve the number of retweets for each tweet ID. Finally, for each headline we determined the total number of shares by summing the number of tweets containing that headline and the number of retweets that each of those tweets received. We also recorded the date of the earliest tweet for each headline, allowing us to calculate the age of the headline.

### Ethical statement

Participants provided informed consent, and our studies were approved by the Yale Human Subject Committee, IRB Protocol # 1307012383, the University of Regina Research Ethics Board, Protocol #2018–116, and by the MIT COUHES, Protocol #1806400195.

## Results

**Fig 1:**
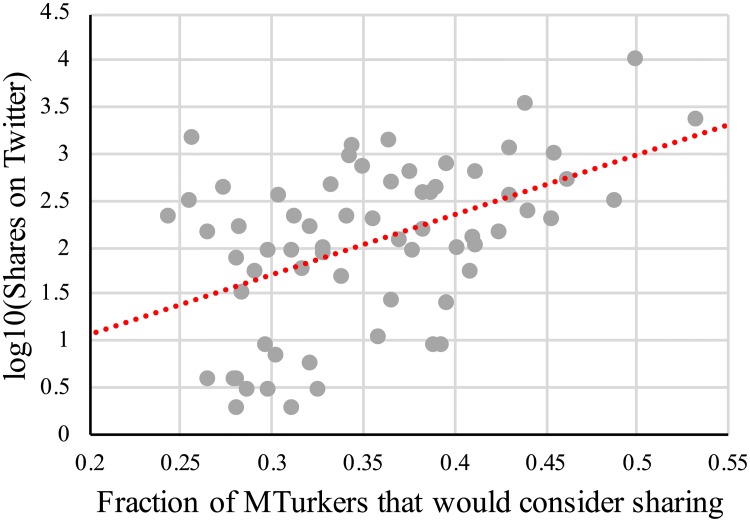
Correlation between fraction of MTurkers who indicated that they would consider sharing a given headline on social media and the number of shares (tweets+retweets) the headline actually received on Twitter.

We begin by considering the correlation between the fraction of MTurk subjects who would consider sharing a headline and that headline’s Twitter sharing count (Because the distribution of Twitter share counts is highly right-skewed, we log10-transform these counts in our correlation analyses.). As predicted, we find a significant and large correlation, *r*(66) = .47, *p* = .0001, such that headlines that MTurkers were more willing to share did indeed receive substantially more shares on Twitter. As can be seen in Fig 1, the relationship is such that across the relevant range of MTurk sharing fractions, a 17 percentage point increase in the fraction of MTurkers who consider sharing a headline is associated with an average of 10x as many actual shares on Twitter. This finding is robust to considering only the number of original tweets, *r*(66) = .43, *p* = .0003, or only the number of retweets, *r*(66) = .46, *p* = .0001; to excluding headlines with 10 or fewer total Twitter shares, *r*(52) = .36, *p* = .0007; and to normalizing number of Twitter shares by the age of the headline (We divide number of shares by age in days, and then log-transform the shares/day value.), *r*(66) = .43, *p* = .0002. We also find similar results in a linear regression controlling for headline veracity when predicting Twitter sharing using the fraction of MTurkers who consider sharing the headline, *β* = .40, *t* = 3.12, *p* = .003. Finally, for 57 of the headlines, we also had a measure of the extent to which participants believed they had seen the headline previously. When including this familiarity measure as a control, as well as headline veracity, we continue to observe a significant positive relationship with the fraction of MTurkers who consider sharing the headline, *β* = .42, *t* = 2.82, *p* = .007.

To further demonstrate the robustness of this association, we use negative binomial regression to predict the number of Twitter shares as a function of *z*-scored MTurk sharing intentions. We find a significant positive relationship, *IRR* = 1.918, *z* = 4.63, *p* < .0001. This finding is again robust to considering only the number of original tweets, *IRR* = 2.27, *z* = 4.65, *p* < .0001, or only the number of retweets, *IRR* = 1.58, *z* = 3.43, *p* = .0006; to excluding headlines with 10 or fewer total Twitter shares, *IRR* = 1.71, *z* = 4.59, *p* < .0001; and to including controls for headline veracity and headline age, *IRR* = 1.77, *z* = 3.04, *p* = .002, or controls for veracity, age, and prior familiarity among the 57 headlines for which familiarity data was available, *IRR* = 1.94, *z* = 3.35, *p* = .001. Equivalent results are also obtained using Poisson regression instead of negative binomial regression.

Additionally, we compared the correlation between various headline characteristics and the likelihood of being shared on MTurk and Twitter. In addition to examining headline veracity, we used the Linguistic Inquiry and Word Count (LIWC; [19]) dictionaries to determine the presence of Emotional, Moral, or Moral-Emotional language (following work by [2]), as well as language related to Religion, Inhibition, and Insight (following work by [20]); and we used the Dale-Chall formula [21] to determine the complexity of the language used. When correlating these characteristics with sharing, we found identical patterns across the Mturk and Twitter sharing data. False headlines were shared less on both MTurk (*r*(66) = -0.536, *p*<0.001) and Twitter (*r*(66) = -.343, *p* = 0.004) compared to true headlines; headlines containing Moral words were shared less on both on MTurk (*r*(66) = -0.275, *p* = 0.023) and Twitter (*r*(66) = -0.317, *p* = 0.009); and there was no significant correlation between any of the other headline characteristics and sharing on either MTurk or Twitter (*p*>0.1 for all). See Table 1 for full list of correlations.

**Table 1:** Correlation between various headline characteristics and the likelihood of being shared on MTurk and Twitter.

	MTurk (z-scored)	Twitter (log-transformed)
Veracity	-0.536 (*p* < .001)	-0.343 (*p* = 0.004)
Emotional language	-0.118 (*p* = 0.337)	-0.076 (*p* = 0.540)
Moral language	-0.275 (*p* = 0.023)	-0.317 (*p* = 0.009)
Moral-Emotional language	0.120 (*p* = 0.331)	0.129 (*p* = 0.295)
Religious language	-0.073 (*p* = 0.554)	-0.025 (*p* = 0.843)
Insight language	-0.173 (*p* = 0.157)	-0.182 (*p* = 0.137)
Inhibition language	-0.051 (*p* = 0.678)	0.098 (*p* = 0.426)
Language complexity	0.160 (*p* = 0.193)	0.026 (*p* = 0.831)

## Discussion

The findings presented here suggest that self-reported sharing intentions collected on Amazon Mechanical Turk are likely to provide reasonable insight into what content would actually be shared on social media. Headlines that more MTurkers reported that they would considering sharing did in fact receive substantially more tweets and retweets on Twitter. This is an encouraging finding for social scientists interested in studying social media sharing in the context of traditional survey experiments.

Of course, there are numerous limitations to the current study. First, we only considered a handful of the almost limitless number of political news headlines that have been posted online. Furthermore, we considered only American subjects and American political news. Relatedly, we considered only political news, and not any of the many other kinds of content that people share online. Thus, it remains to be seen whether our results will generalize to other news headlines, countries, or types of content. Second, there are many factors that contribute to the number of (re)tweets a story receives on Twitter which are missing from our survey experiment. For example, the activity of bots; the social context of who shared the news story; and the sociopolitical context of the specific time at which the story was posted. If anything, however, the absence of these factors would deflate the observed correlation–thus our estimate provides a lower bound. Third, for logistical and privacy reasons, our analyses were conducted at the level of the headline rather than the individual. Thus, we did not observe whether the specific individuals who say they would share a given headline actually share that headline. As a result, we cannot draw conclusions about how intentions predict sharing at the *individual* level. Thus, future work should investigate the link between self-reported and actual sharing at the level of the individual. Be that as it may, however, many of the important questions in this space are in fact headline-level questions (e.g. how likely are false headlines to shared?). Fourth, the fact that we observe a substantial correlation between unmanipulated sharing intentions on MTurk and actual sharing on Twitter does not necessarily mean that the latter will respond to experimental manipulations in the same way as the former (as in, for example, [22])—for example, it is possible that interventions which successfully affect MTurk sharing intentions would not be effective on Twitter because of the numerous differences in the context in which the participants are making their decisions. Fifth, the MTurk data was collected at the end of the time period (2019) during which the Twitter data was collected (2016–2019). This means that MTurk respondents could be more likely to share news items that are more popular simply because those headlines have grown in popularity over time on social media. This seems unlikely, however, for two reasons: popularity on social media tends to be extremely ephemeral, and experimental evidence suggests that being familiar with a story actually makes people less likely to say they would share it, rather than more likely [13]. Finally, our samples from MTurk users are not necessarily representative of Twitter users. Although our headline-level analysis suggests a meaningful relationship between the likelihood of sharing between the two crowds, future work should study how these results generalize to self-reported measures collected from other crowds.

We hope that the results reported here will contribute to the increasing study of online behavior by psychologists and other social scientists, and will inspire future individual-level methodological work on the validity of self-reported sharing intentions. Much of our social life is now conducted online, and understanding what motives sharing decisions on social media is an extremely important area for study using the lens of behavioral science.
